# Fluorescently Labeled Methyl-Beta-Cyclodextrin Enters Intestinal Epithelial Caco-2 Cells by Fluid-Phase Endocytosis

**DOI:** 10.1371/journal.pone.0084856

**Published:** 2014-01-08

**Authors:** Ferenc Fenyvesi, Katalin Réti-Nagy, Zsolt Bacsó, Zsuzsanna Gutay-Tóth, Milo Malanga, Éva Fenyvesi, Lajos Szente, Judit Váradi, Zoltán Ujhelyi, Pálma Fehér, Gábor Szabó, Miklós Vecsernyés, Ildikó Bácskay

**Affiliations:** 1 Department of Pharmaceutical Technology, University of Debrecen, Debrecen, Hungary; 2 Department of Biophysics and Cell Biology, University of Debrecen, Debrecen, Hungary; 3 Cyclolab Cyclodextrin R&D Laboratory Ltd., Budapest, Hungary; University of Szeged, Hungary

## Abstract

Cyclodextrins are widely used excipients for increasing the bioavailability of poorly water-soluble drugs. Their effect on drug absorption in the gastrointestinal tract is explained by their solubility- and permeability-enhancement. The aims of this study were to investigate penetration properties of fluorescently labeled randomly methylated-beta-cyclodextrin (FITC-RAMEB) on Caco-2 cell layer and examine the cellular entry of cyclodextrins on intestinal cells. The permeability of FITC-RAMEB through Caco-2 monolayers was very limited. Using this compound in 0.05 mM concentration the permeability coefficient was 3.35±1.29×10^−8^ cm/s and its permeability did not change in the presence of 5 mM randomly methylated-beta-cyclodextrin. Despite of the low permeability, cellular accumulation of FITC-RAMEB in cytoplasmic vesicles was significant and showed strong time and concentration dependence, similar to the characteristics of the macropinocytosis marker Lucifer Yellow. The internalization process was fully inhibited at 0°C and it was drastically reduced at 37°C applying rottlerin, an inhibitor of macropinocytosis. Notably, FITC-RAMEB colocalized with the early endosome organizer Rab5a. These results have revealed that FITC-RAMEB is able to enter intestinal epithelial cells by fluid-phase endocytosis from the apical side. This mechanism can be an additional process which helps to overcome the intestinal barrier and contributes to the bioavailability enhancement of cyclodextrins.

## Introduction

Cyclodextrins are water-soluble cyclic oligosaccharides with hydrophilic outer surface and hydrophobic inner cavity. Their chemical structure enables them to form inclusion complexes with lipophilic molecules in aqueous solutions leading to the increment of aqueous solubility of guest molecules. The complex formation ability of cyclodextrins is utilized mainly in pharmaceutical industry for the formulation of water insoluble or poorly soluble drugs of Class II and Class IV of the Biopharmaceutics Classification System (BCS). Solubility- and absorption-enhancing effects of cyclodextrins lead to higher bioavailability of intestinal formulations, and complex formation can increase the stability of active substances [1] [2]. Several cyclodextrin derivatives were synthesized to improve the complexation efficacy and decrease toxicity. Lipophilic cyclodextrins such as methylated cyclodextrins (e.g. randomly methylated β-cyclodextrin) and hydrophilic cyclodextrins like hydroxypropyl derivatives (e.g. 2-hydroxypropyl-β-cyclodextrin) are distinguished, even if their solubility in water is high [3]. Besides the pharmaceutical applications, β-cyclodextrins are also used in cell biology research for the removal of cholesterol from cell membrane [4] and to study the role of cholesterol on cellular functions. In the case of β-cyclodextrins a relationship could be identified among the substituents of the cyclodextrin ring, cholesterol solubilization, hemolytic activity and cytotoxicity [5]. Membrane cholesterol extraction can induce several cellular effects. The activity of membrane transporters, such as P-glycoprotein is sensitive to the presence of cholesterol [6], [7], [8]. The disruption of cholesterol rich membrane rafts alters the integrity of tight junctions and barrier functions of cell layers [9], [10]. These effects can also increase the permeability and absorption of drug molecules from the intestine. On the other hand membrane cholesterol depletion with high cyclodextrin concentration inhibits endocytotic processes [11], [12] and increases exocytosis [13].

The chemical structure, number of hydrogen donors and acceptors, relatively high molecular weight (>1000 Da) and the hydrophilicity of cyclodextrins predict that these molecules are not able to permeate biological membranes and have poor absorption [14]; only lipophilic cyclodextrins are considered to be absorbed from the gastrointestinal tract to some extent [3]. In general, only the free form of drug, which dissociates from the cyclodextrin complex, is thought to be absorbed. According to this mechanism cyclodextrin delivers the drug to the surface of cell membrane, the drug molecule penetrates into the lipophilic membrane, but after delivery the cyclodextrin remains extracellular [3]. Interestingly in vivo studies showed that relatively high amount of hydroxypropyl-β-cyclodextrin and dimethyl-β-cyclodextrin were absorbed via rectum of rats and excreted into the urine, suggesting that not only the free form of drugs, but also cyclodextrin complexes may be absorbable through the rectal mucosa [15].

Although cyclodextrins most likely cannot permeate the cell membrane by diffusion, recent findings revealed that they are able to enter cells. Methyl-β-cyclodextrin-dextran conjugates and hydroxypropyl-β-cyclodextrin were found to enter cells by endocytosis, as they reduced intracellular cholesterol accumulation in Niemann-Pick type C mutant cells acting at the level of endocytotic organelles inside the cells [16]. Intracellular accumulation of the fluorescent mono-4-(N-6-deoxy-6-amino-β-cyclodextrin)-7-nitrobenzofuran (NBD-β-CD) was also detected in HepG2 and SK-MEL-24 cells, and endocytosis as a possible mechanism for the transmembrane passage of NBD-β-CD was suggested [17]. Macropinocytosis of amphiphilic cationic cyclodextrin transfection complexes were also observed in Caco-2 intestinal epithelial cells [12], and clathrin-dependent endocytosis of a fluorescent methyl-β-cyclodextrin by HeLa cells was demonstrated [18].

These results raise the possibility that cyclodextrin molecules not only increase the solubility of poorly soluble drugs and act as permeation enhancers in the intestine, but are able to enter intestinal cells by the endocytotic pathway. This mechanism, the intracellular route and fate of cyclodextrins have not been investigated on intestinal epithelial cells yet, although transcytosis is known in the case of intestinal epithelial Caco-2 cells [19]. There is also limited information about the permeability of cyclodextrins on Caco-2 monolayers.

In the present study our aim was to examine the interaction of the fluorescently labeled randomly methylated β-cyclodextrin (FITC-RAMEB) with Caco-2 colon cell layer and examine the cellular uptake of cyclodextrins on intestinal epithelial cells.

## Materials and Methods

Randomly-methylated β-cyclodextrin (RAMEB) was purchased from Wacker Chemie (Munich, Germany). 6-monodeoxy-6-mono[(5/6)-fluoresceinylthioureido]-RAMEB (FITC-RAMEB) (DS = 1 for FITC, DS ∼ 12 for methyl) was the product of CycloLab Ltd(Budapest, Hungary). FITC-RAMEB was prepared by reacting methylated amino-cyclodextrin with fluorescein-5(6)-isothiocyanate as described elsewhere [18]. By this reaction FITC was covalently coupled to RAMEB. CellMask Deep Red plasma membrane stain and CellLight^®^ Early Endosomes-RFP *BacMam 2.0* was from Invitrogen (Budapest, Hungary). All other reagents were purchased from Sigma-Aldrich (Budapest, Hungary).

### Caco-2 Cell Culture

Caco-2 cell line originates from the European Collection of Cell Cultures (ECACC UK). Caco-2 cells were cultured in Dulbecco’s modified Eagle’s medium (DMEM) supplemented with 10% heat-inactivated foetal bovine serum, 1% non essential amino acid and 1% penicillin-streptomycin solution at 37°C in an incubator containing 5% CO_2_. The passage number of the cells was between 25 and 40.

For permeability experiments and release studies, Caco-2 cells were seeded at density of 200,000 cells/well on Transwell® (Corning Costar, USA) polycarbonate filters (pore size 0.4 µm, surface area 1.12 cm^2^). Culture medium was replaced with fresh medium every two or three days in the inserts. Monolayers were used for the experiments between 20 and 35 days after seeding. The formation of functional epithelial layers was monitored by the development of transepithelial electrical resistance (TEER) and measured with a Millicell–ERS voltohmmeter (Millipore, USA). In permeability experiments TEER values were also measured at the beginning and at the end of sampling to check monolayer integrity and follow the effects of cyclodextrin treatments.

### Transepithelial RAMEB Permeability Measurements

In permeability measurements two different cyclodextrin solutions were used for the treatments: 0.05 mM FITC-RAMEB (FR) solution and 5.0 mM RAMEB solution containing 0.05 mM FITC-RAMEB (FRR). The solvent was Hank’s Balanced Salt Solution (HBSS). Caco-2 monolayers were washed twice and pre-incubated with HBSS for 20 minutes at 37°C and then incubated apically with FR or FRR solutions for 2 hours at 37°C. Samples were collected from the basolateral side at 60 and 120 minutes and the volume was replenished with HBSS. The monolayers were washed five times with HBSS and cells were lysed with 1% Triton X-100 (TX-100) (Roche Diagnostics GmbH (Mannheim, Germany). The permeated amount and the FITC-RAMEB content of the cell lysates were determined by FLUOstar Optima microplate reader (BMG LABTECH, Offenburg, Germany) at 492 nm excitation and 520 nm emission wavelength.

FITC-RAMEB permeation rates across the monolayers were determined from the concentration values. With the formula below the apparent permeability coefficients were calculated:
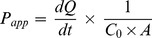



P_app_: apparent permeability coefficient (cm/s)

dQ/dt: permeability rate of substances (mol/s)

C_0_: initial concentration of the substances in the apical chamber (mol/ml)

A: surface area of the membrane (cm^2^).

### FITC-RAMEB Uptake Studies of Caco-2 Monolayers

In this experiment Caco-2 cells were seeded in black 96 well plates at the density of 10^4^ cells/well. After 7 days the cells were washed twice with HBSS and incubated with FR or FRR solutions at 37°C for 5-, 10-, 30-, 60- or 120 minutes. After the treatment cells were washed four times with ice cold HBSS, kept on ice and fixed with 3% paraformaldehyde solution (37°C, 15 min). Fluorescence intensities of the samples were measured with FLUOstar Optima microplate reader. After the measurement 4′,6-diamidino-2-phenylindole dihydrochloride (DAPI), at 300 nM final concentration was added to each well and incubated for further 15 minutes. DAPI was measured at 355 nm excitation and 485 nm emission wavelengths. This dye was used to normalize FITC-RAMEB for DAPI fluorescence intensities.

### FITC-RAMEB Release Studies on Caco-2 Monolayers

The first part of this experiment was the same like permeability measurements, except that the apical chamber contained 0.5 mM FITC-RAMEB solution. At the end of the 120 minutes long incubation, inserts were washed five times with ice cold HBSS and divided into two groups. The monolayers of the control group were lysed with 1% Triton X-100 solution and FITC-RAMEB contents were determined with FLUOstar Optima microplate reader. The other group of the inserts was incubated in HBSS at 37°C for another 120 minutes. During the second incubation samples were collected from apical and basolateral chambers at 10-, 30-, 60- and 120 minutes and the released amount of FITC-RAMEB was measured. After incubation these monolayers were also washed twice with HBSS, lysed and the FITC-RAMEB content of the cell layers was determined. The rate of the release was expressed as the percentage of FITC-RAMEB content of the monolayers of control group.

### Confocal Microscopy

For microscopic investigations 80,000 cells/well were seeded on round glass cover-slips in 12-well plates. 24 hours later cells were treated with CellLight^®^ Early Endosomes-RFP *BacMam 2.0* at density of 30 particles per cell and incubated for further 48 hours in cell culture medium. Then samples were washed twice with HBSS and treated with FR or FRR solutions for 30 minutes at 37°C. To completely remove FITC-RAMEB, cells were washed eight times with ice cold HBSS and samples were stained with 1 µg/ml solution of CellMask Deep Red plasma membrane stain for 5 minutes at 37°C. After washing cells twice with HBSS and fixing them with 3% paraformaldehyde solution, cell nuclei were stained with DAPI (300 nM). In some experiments Caco-2 monolayers were treated applying the same protocol for plasma membrane and cell nucleus staining but in these samples Early Endosomes-RFP was not used. In experiments performed in Transwell®, the insert membranes were excised and placed on slides. Confocal microscopy measurements and analyses were carried out by a Zeiss LSM 510 META (Jena, Germany) confocal microscope. To eliminate spectral cross talk samples were illuminated with three different excitations subsequently using multi-track mode (UV lines: 351.1 nm and 363.8 nm of an Ar-ion laser, these two lines were used simultaneously; blue line: 488 nm of another Ar-ion laser; and red line: 633 nm of a He-Ne laser). Emissions above 420 nm, above 505 nm and above 650 nm were detected subsequently in three channels with the META detector, respectively. For confocal imaging pinhole size was set to 1 Airy unit.

### Flow Cytometry

Flow cytometric experiments were used to verify endocytosis of FITC-RAMEB by Caco-2 cells. For these experiments cells were trypsinized, washed twice with HBSS and resuspended at 1×10^6^ cells/ml concentration. Cells were incubated with FITC-RAMEB, Lucifer Yellow (LY) or calcein AM solutions in different concentrations for 30 minutes at 37°C or at 0°C. Dyes were used in the following concentration ranges: FITC-RAMEB from 0 to 500 µM, calcein AM from 0 to 1 µM, and LY from 0 to 960 µM. At the end of the treatments cells were washed three times with ice cold HBSS and kept on ice until measurements. Propidium-iodide was added to the cells at the concentration of 2 µg/ml to recognize dead cells. In uptake inhibition experiments cells were pre-incubated with 10 µM rottlerin for 45 minutes before adding FITC-RAMEB or LY. Cells were analyzed by five-laser BD FACSaria II flow cytometer (BD Biosciences, San Jose, CA). In the case of FITC-RAMEB and calcein AM staining, cells were illuminated with 488 nm laser line, while for LY staining with the more optimal 445 nm. In all previous cases fluorescence emission was detected via 502 nm long pass dichroic mirror and 530/30 nm band pass filter. Single cell events were recognized using both the area and width of the forward-scattered light and side-scattered light signals. Viable cells were gated in according to their low intensity propidium iodine fluorescence excited at 561 nm and detected via 590 nm long pass filter.

### Statistical Analysis

For statistical analysis SigmaStat softver (version 3.1; SPSS Inc.) was used. Data are presented as means ± SD. Comparison of two groups was performed by unpaired or paired t-test, while comparison of more than two groups was performed using ANOVA. Differences were considered significant at p<0.05.

## Results

### Transepithelial FITC-RAMEB Permeability in Caco-2 Cell Monolayers

In order to investigate the permeability of the fluorescent derivative of RAMEB through the intestinal epithelial barrier we applied Caco-2 monolayers. Two cyclodextrin solutions, 0.05 mM FITC-RAMEB (FR) and 5.0 mM RAMEB solution containing 0.05 mM FITC-RAMEB (FRR) were used. The permeability of FITC-RAMEB was determined in both cases and the results were expressed in apparent permeability values (P_app_). The apparent permeability of FITC-RAMEB was very low both in FR and FRR treatments, 3.35±1.29×10^−8^ and 4.23±1.46×10^−8^ cm/s, respectively. There was no significant difference between these two average permeability values (n = 9 for FR and n = 6 for FRR treatments, p>0.05), indicating that 5 mM RAMEB co-treatment had no effect on the permeability of FITC-RAMEB and the integrity of the monolayer.

**Figure 1:**
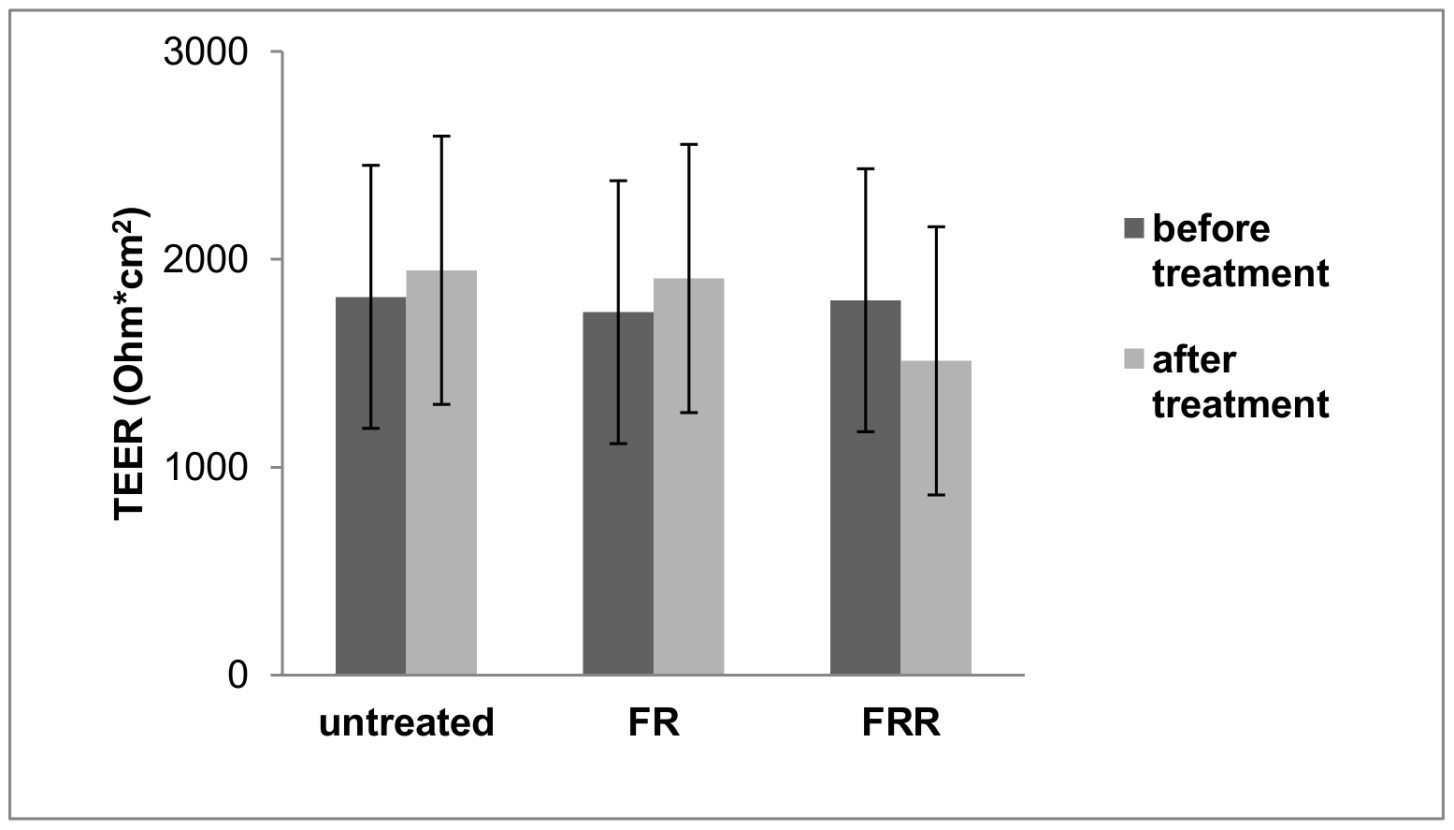
Transepithelial electric resistance (TEER) of Caco-2 monolayers before and after 120 minutes permeability experiments. Cell layers were treated with 0.05-RAMEB (FR) alone or in the presence of 5 mM RAMEB (FRR). Untreated monolayers were kept in HBSS. Values are expressed as means ± SD, n = 9 for FR, n = 6 for FRR treatment and n = 6 for untreated samples. There were no significant differences between TEER values before and after the treatments (p>0.05) and among the groups (p>0.05).

The integrity of monolayers was tested by measuring transepithelial electrical resistance (TEER). The TEER values did not decrease significantly after the cyclodextrin treatments (p>0.05) (Fig. 1).

**Figure 2:**
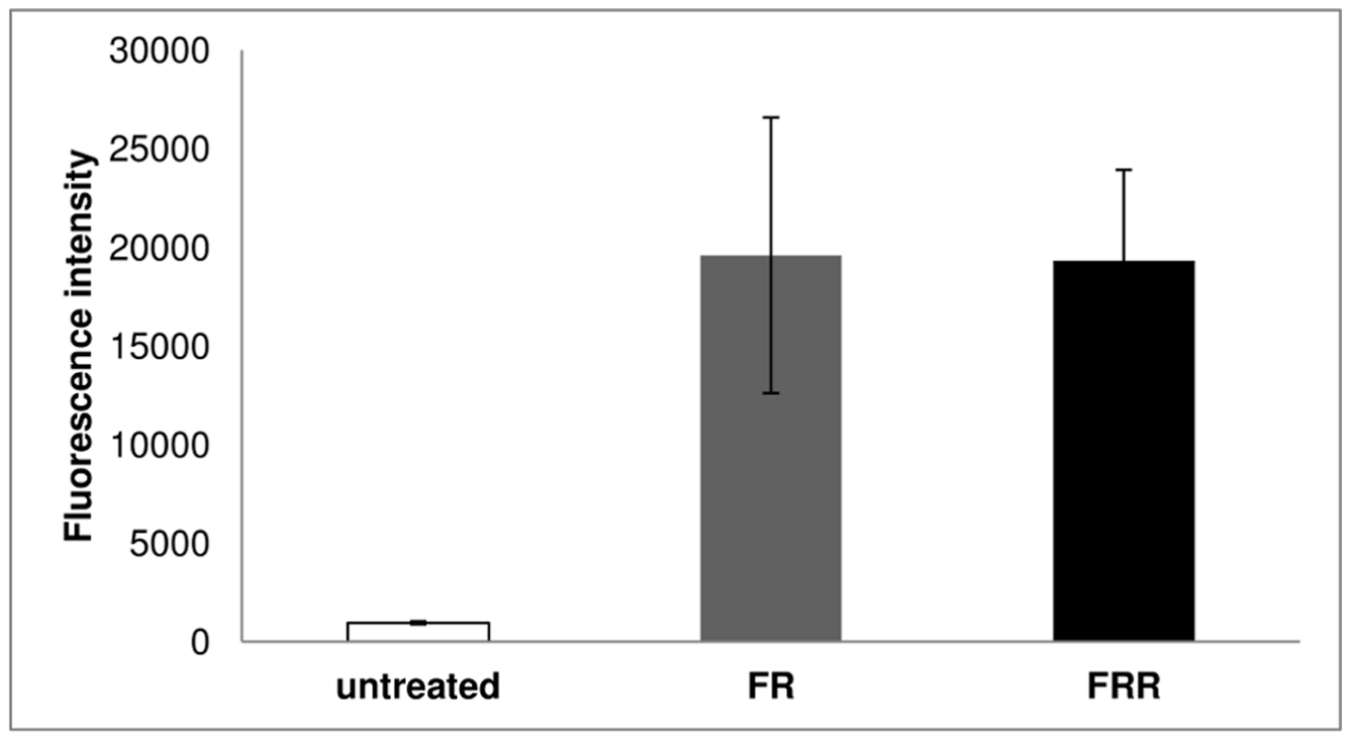
Accumulation of FITC-RAMEB in Caco-2 monolayers after 120 minutes permeability experiments. Caco-2 monolayers were treated with 0.05 mM FITC-RAMEB (FR) alone or in combination with 5 mM RAMEB (FRR) for 120 minutes. Monolayers were washed and the fluorescence intensity of the accumulated FITC-RAMEB was determined with FLUOstar Optima microplate reader. Presented values are means ± SD, n = 7 for FR, n = 4 for FRR treatment and n = 5 for untreated samples. FR and FRR treatments increased significantly the fluorescence of monolayers compared to the untreated control (p<0.001).

At the end of the permeability measurements cell layers were washed thoroughly with HBSS and lysed with 1% TX-100. The fluorescence of cell lysates were measured with FLUOstar Optima microplate reader. The fluorescence of FR and FRR treated samples were significantly higher than the untreated monolayers (p<0.001), indicating, that Caco-2 cell layers accumulated fluorescently-labeled RAMEB (Fig. 2). FRR, containing 5 mM RAMEB did not change the accumulation of FITC-RAMEB in the cell layers (p>0.05).

### FITC-RAMEB Accumulation in Caco-2 Monolayers

**Figure 3:**
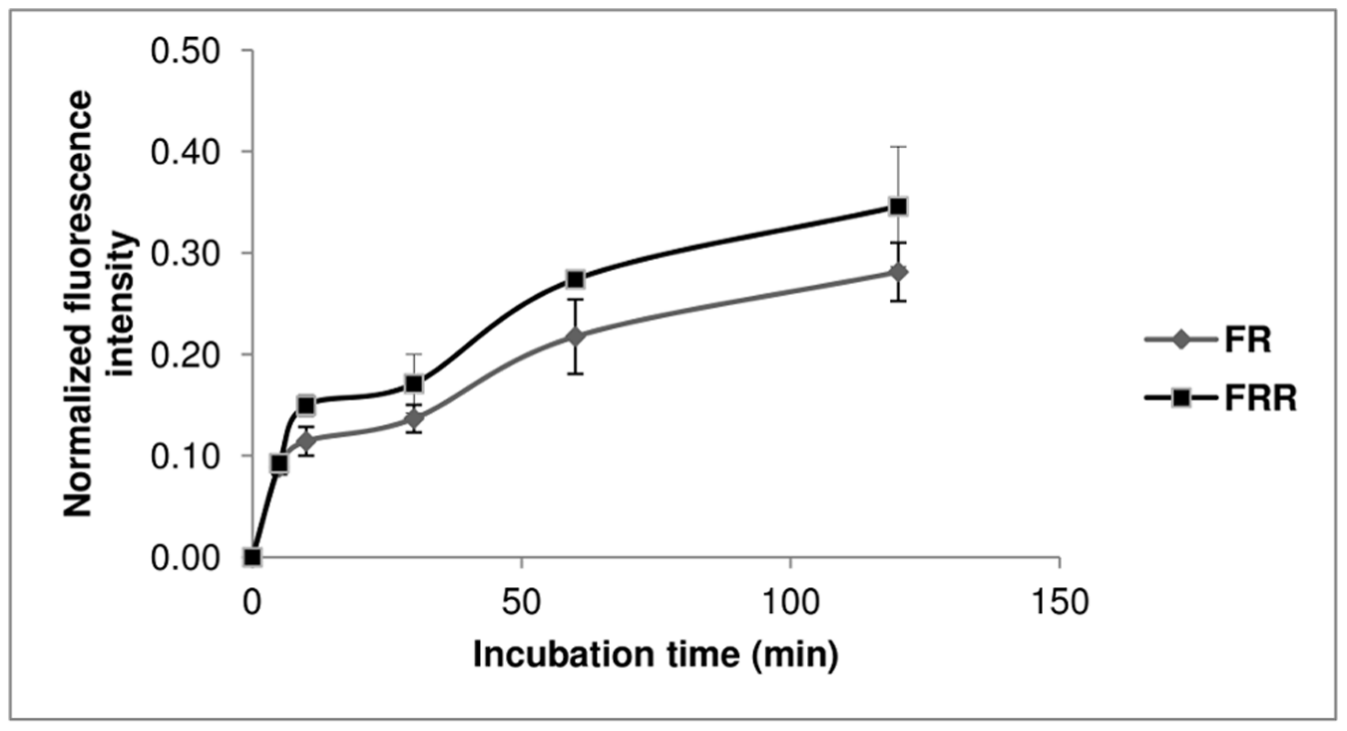
Kinetics of FITC-RAMEB uptake in Caco-2 monolayers. Cell monolayers were treated with 0.05-RAMEB (FR) alone or in combination with 5 mM RAMEB (FRR) and in different time points the incubation was stopped. After washing cells were fixed with 3% paraformaldehyde solution and the accumulated FITC-RAMEB was determined by microplate reader. Cell nuclei were labeled with DAPI and fluorescence intensities of FITC-RAMEB were normalized for DAPI fluorescence intensities. Values are expressed as means ± SD, n = 3 for FR and FRR treatments.

The time dependence of FITC-RAMEB accumulation was measured in 96-well plates with a microplate reader. Caco-2 cells were treated with FR and FRR solutions for 5-, 10-, 30-, 60- or 120 minutes. No difference could be seen between FR and FRR treatment up to 120 minutes. The accumulated amount of FITC-RAMEB increased during the 120 minutes of the experiment. The rate of uptake was fast during the first 5–10 minutes and slower in the remaining period (Fig. 3).

### Release of Accumulated FITC-RAMEB from Caco-2 Monolayers

**Figure 4:**
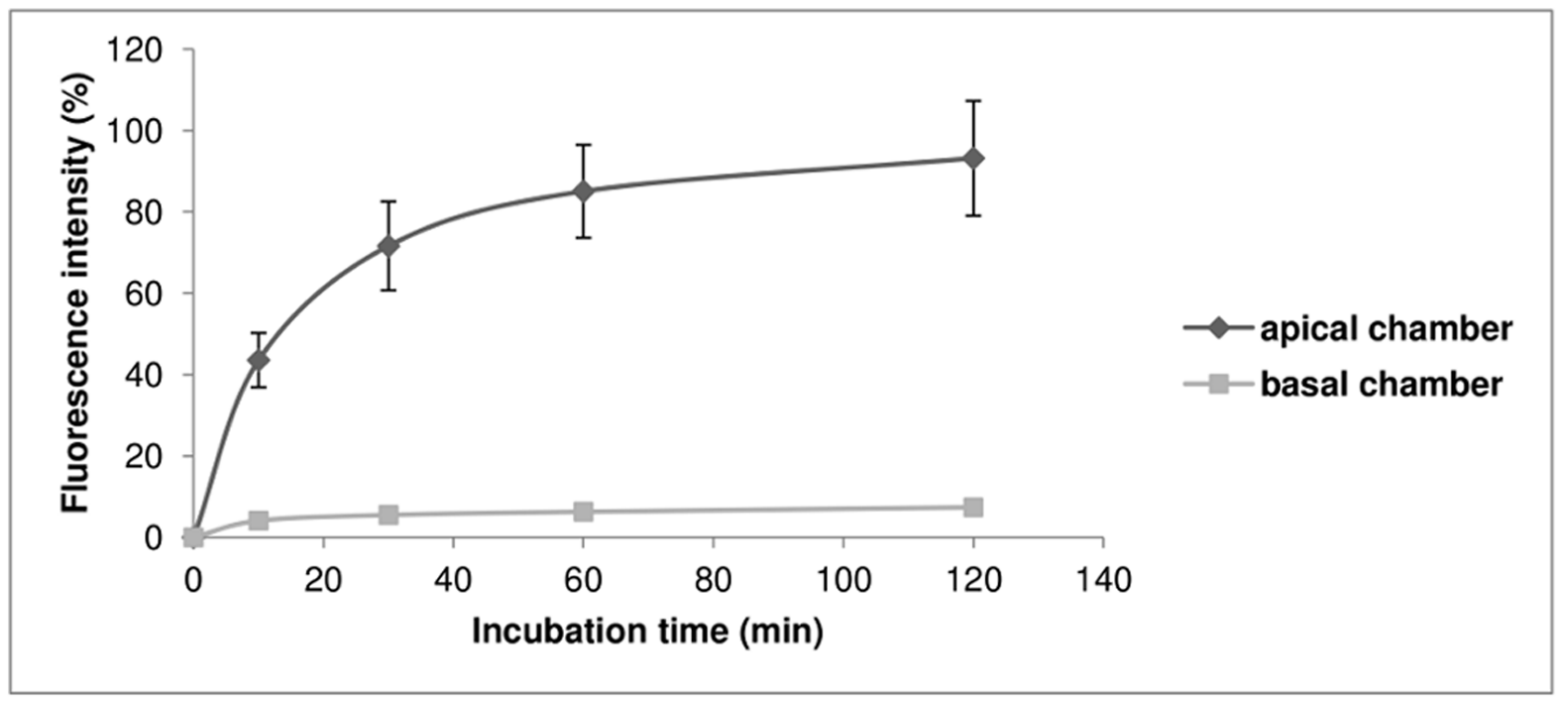
Release of FITC-RAMEB from Caco-2 monolayers. Treatment with 0.5-RAMEB was carried out on Transwell® inserts for 120 minutes, then the monolayers were washed and FITC-RAMEB release was followed in the apical and basolateral chambers during the next 120 minutes. FITC fluorescence intensities were determined in the control group of the samples after the first 120 minutes and considered as 100% accumulation. The rate of release in the second group was compared to this value. Values are means ± SD, n = 4.

In FITC-RAMEB release studies Caco-2 monolayers were treated with 0.5 mM FITC-RAMEB solution for 120 minutes, washed five times with ice-cold HBSS and the fluorescence of control group of monolayers was determined and considered as 100%. The second group of monolayers was kept for further 120 minutes in fresh HBSS at 37°C and the released amount of FITC-RAMEB in apical and basal chambers was determined as a function of time. FITC-RAMEB appeared rapidly in both chambers. About 85% of initial fluorescence was released into the apical chamber within the first hour, while only about 7.4% of the accumulated FITC-RAMEB was released into the basal chamber from monolayers after 2 hours of incubation (Fig. 4). At the same time the fluorescence of the monolayers decreased drastically, from 100±8.8% to 8.9±1.5%.

### FITC-RAMEB Internalization in Undifferentiated and Differentiated Caco-2 Cells and Colocalization with Rab5a Early Endosome Marker

**Figure 5:**
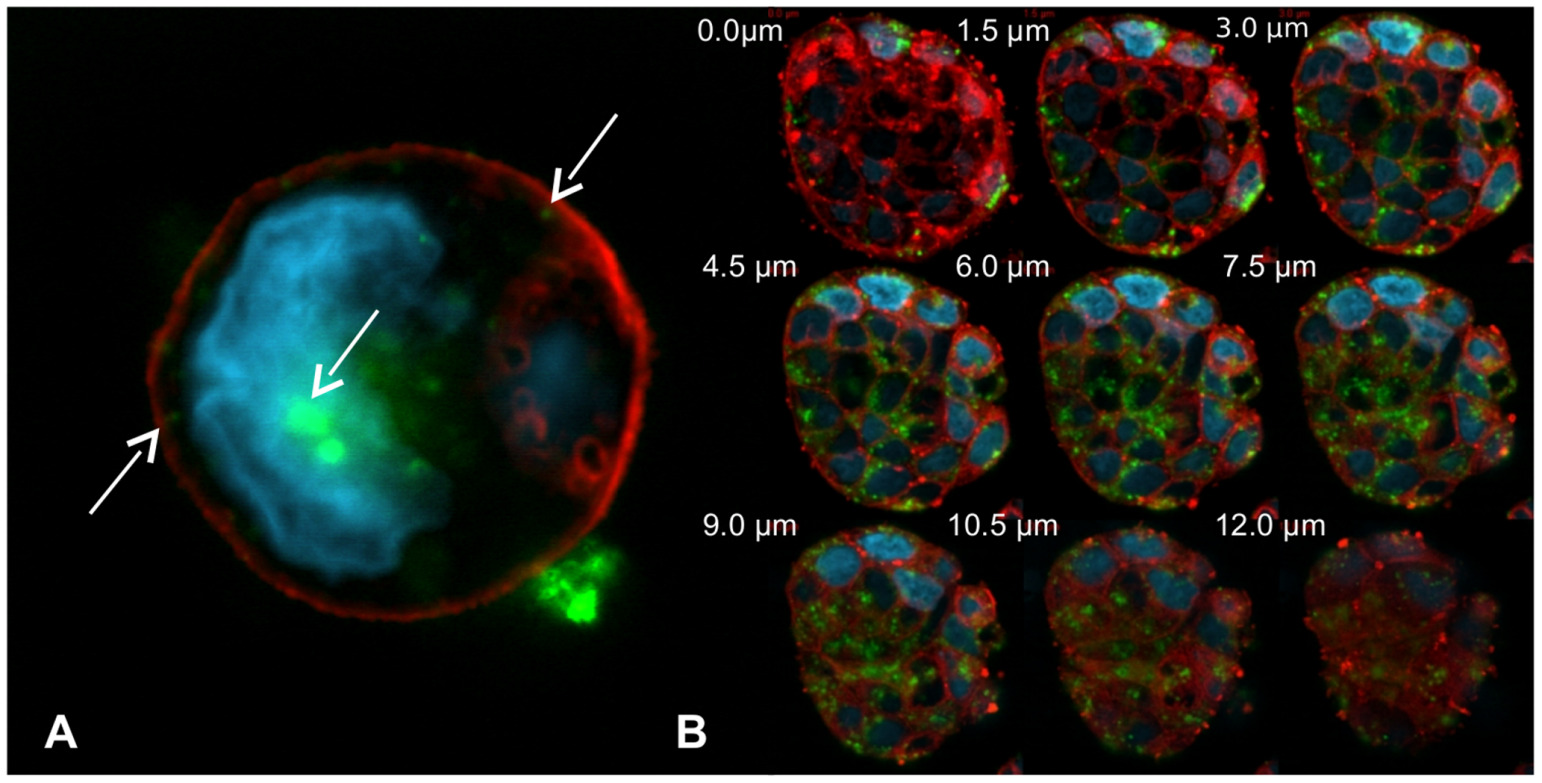
Confocal images of undifferentiated Caco-2 cells. Cells were treated with the solution of 0.05-RAMEB and 5 mM RAMEB (FRR). FITC-RAMEB (green) is localized in small vesicles (white arrows) under the CellMask labeled cell membrane (red) or in larger vesicles near the DAPI stained cell nucleus (light blue). Aggregated particles of FITC-RAMEB can be also seen outside the cell membrane (A). Nine consecutive confocal sections of a cluster of cells were recorded (B). Each section is one and half micrometer thick. FITC-RAMEB (green) is located in cytoplasmic granules. The granular bright particles are observed inside the cell membrane and outside of the cell nuclei.

The accumulation of FITC-RAMEB in Caco-2 cells and monolayers was visualized by confocal laser scanning microscopy. In undifferentiated Caco-2 cells, FITC-RAMEB could be detected on CLSM images as small bright particles, located in the cytoplasm (Fig. 5). In the mid-sections the cyclodextrin-loaded granules were found under the cell membrane and near the cell nuclei.

**Figure 6:**
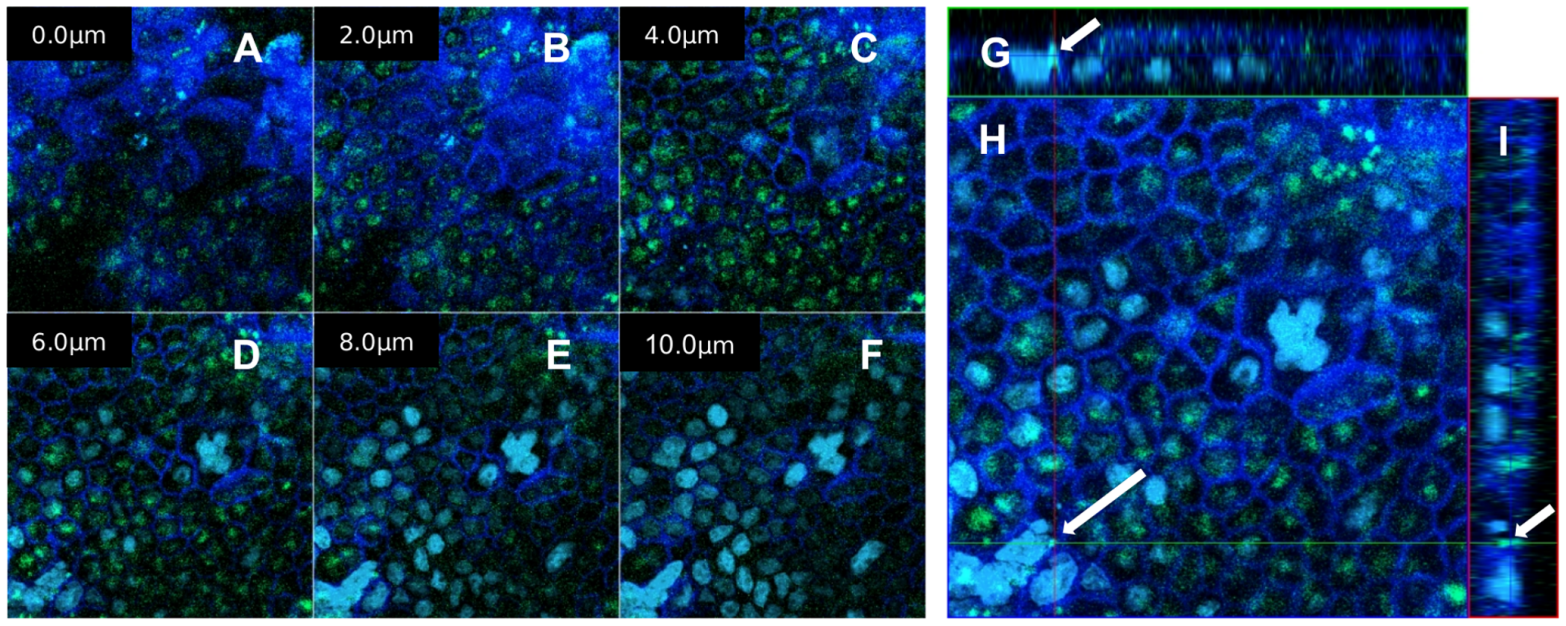
Cyclodextrin enters differentiated Caco-2 cells of a high resistance Caco-2 cell layer. A confluent layer was treated with 0.05-RAMEB and imaged by confocal microscope in twelve two-micrometer thick sections, of which six are demonstrated in the panels on the left side (A–F). On the right, one middle section of the image shows the top view of the cell layer (H) at the level indicated by blue lines in side sections. Upper (G) and right (I) side images are appropriate sections from perpendicular directions at green and at red lines. Crosshair (green and red lines at the long white arrow) set to an intense FITC-RAMEB (green) granule (indicated by arrows), which is located at the nuclear (light blue DAPI stain) level of cells. Several smaller FITC-RAMEB green granules can be seen below the cell membrane marked by CellMask (dark blue).

To examine the FITC-RAMEB uptake of differentiated Caco-2 cells, monolayers grown on Transwell® inserts were used. Fig. 6 shows that FITC-RAMEB is able to enter the Caco-2 monolayer and it is localized in granules within the cytoplasm. There was no difference between cellular uptakes of FITC-RAMEB after FR or FRR treatments on the confocal images.

**Figure 7:**
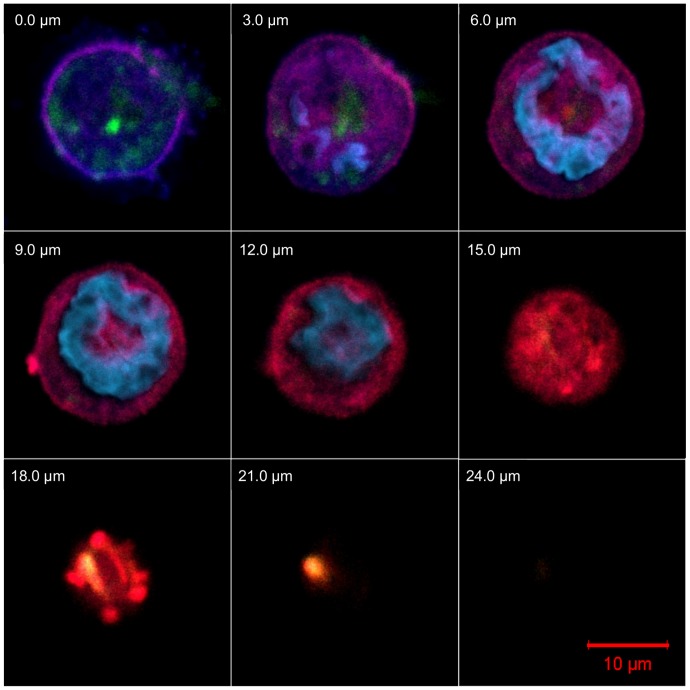
FITC-RAMEB colocalized with Rab5 proteins. RFP-Rab5a transfected Caco-2 cells were treated with 0.05 mM FITC-RAMEB (green) for 30 minutes. Colocalization is indicated by yellow pixels in the confocal microscopic images on the sections marked by the 18-micrometer and 21-micrometer label. Figure shows nine subsequent, three-micrometer thick confocal sections giving altogether a twenty-four-micrometer cross layer of a Caco-2 cell. CellMask (dark blue) was used for labeling membrane at the cell surface and nucleus was stained by DAPI (light blue). Rab5 proteins (red) are visualized by transient transfection of a plasmid coding Red Fluorescent Protein (RFP) tagged Rab5.

These observations suggested that cyclodextrin molecules enter the cells by endocytosis. To confirm this hypothesis, we investigated the colocalization of FITC-RAMEB with the small GTPase Rab5, which is a key determinant of early endosomes [20]. Caco-2 cells were transiently transfected with a plasmid coding for a red fluorescent protein tagged Rab5a GTPase fusion protein (RFP-Rab5a) that was strongly expressed in the cell membrane. As Fig. 7 shows, FITC-RAMEB colocalizes with RFP-Rab5a. Colocalization is marked by yellow pixels. Pearson’s correlation coefficients were calculated and they were between 0.55 and 0.78 after 30 minutes incubation, indicating that the entry of RAMEB into the cytoplasm and the formation of early endosomes are associated. High degree of colocalization could be observed after 2 minutes of incubation, and after 30 minutes colocalization could be still detected indicating, that endocytosis functioned continously (see also Figure S1 and S2.).

### Internalization of FITC-RAMEB and its Inhibition by the Fluid Phase Endocytosis Inhibitor Rottlerin

**Figure 8:**
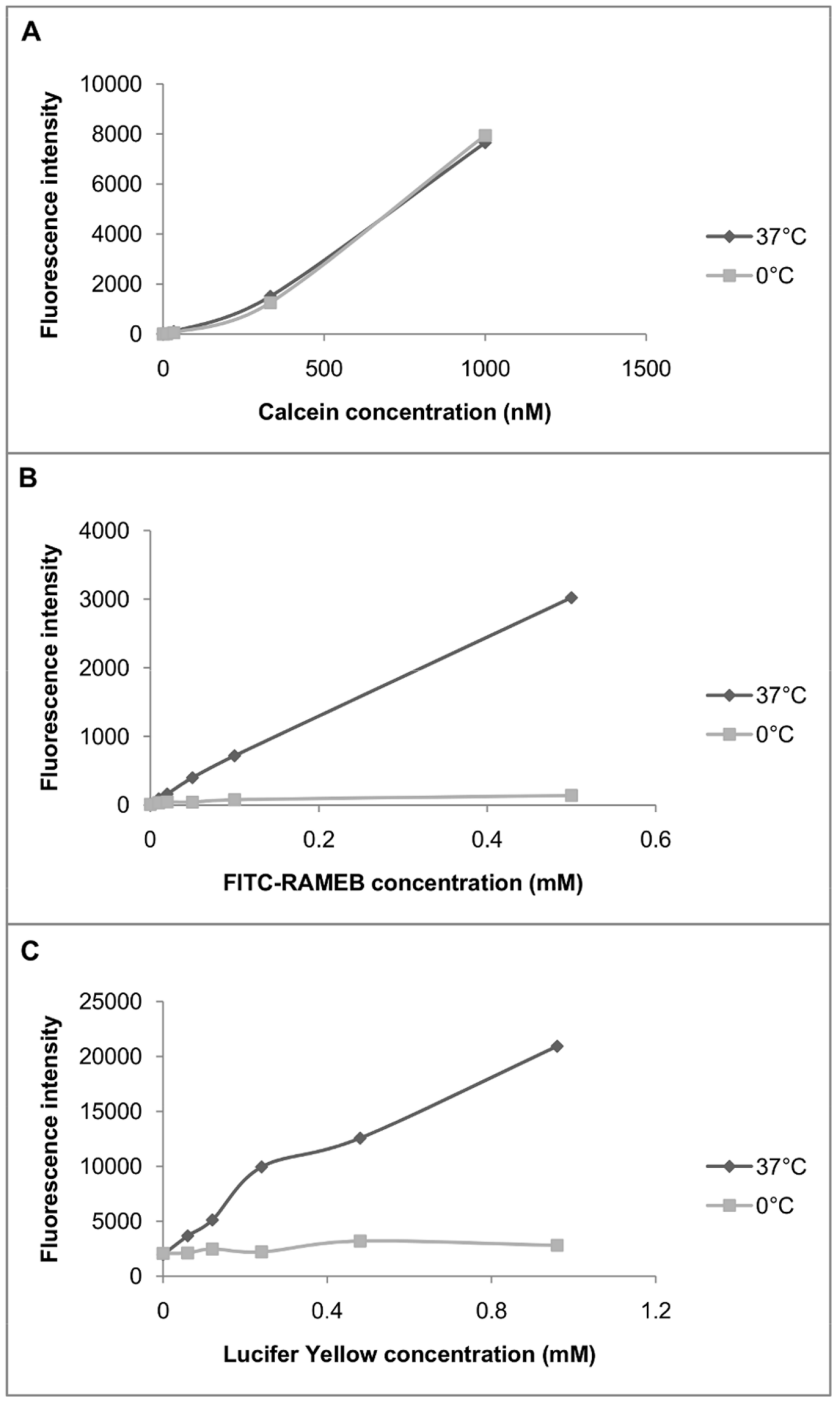
Cellular uptake of calcein-AM (A), FITC-RAMEB (B) and Lucifer Yellow (C) as a function of ligand concentration. Cells were treated at 37°C and 0°C and the cellular fluorescence was determined by flow cytometry, after excluding dead cells with propidium iodide.(Graphs show results of a representative experiment).

Internalization of FITC-RAMEB was also investigated by flow cytometry. Caco-2 cell suspensions were treated by FITC-RAMEB, the macropinocytosis marker Lucifer Yellow and the lipophilic membrane permeability marker calcein-AM, both at 37°C and 0°C. Major differences could be seen between the uptake of hydrophilic and lipophilic molecules (Fig. 8). Intracellular accumulation of calcein was dependent on dye concentration, but was independent of temperature. At the same time, both FITC-RAMEB and Lucifer Yellow uptake increased as a function of the dye concentration, but it was inhibited at 0°C.

**Figure 9:**
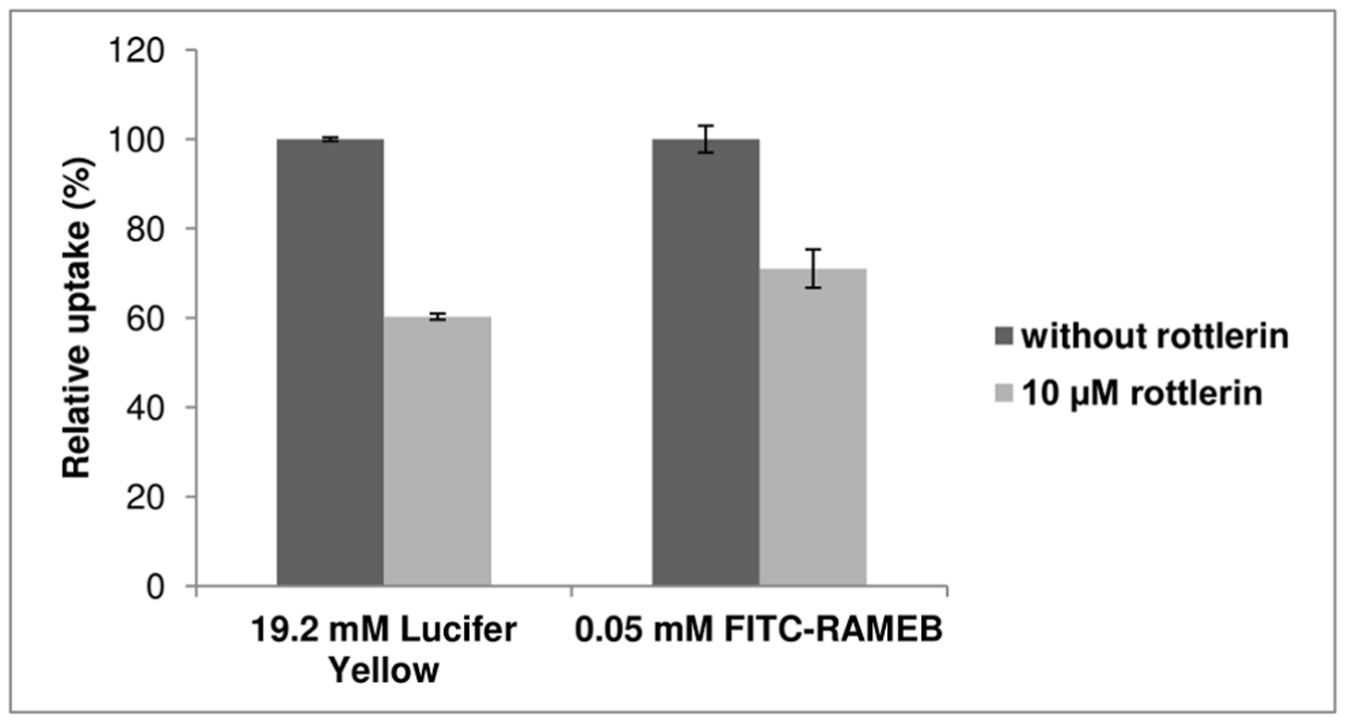
Effect of 10 µM rottlerin on the cellular uptake of FITC-RAMEB and Lucifer Yellow. Caco-2 cells were pre-incubated for 45 minutes with rottlerin and the internalization of the fluorescent molecules was detected by flow cytometer. (n = 3, p<0.01).

Rottlerin, a macropinocytosis inhibitor decreased significantly both FITC-RAMEB and Lucifer Yellow internalization in Caco-2 cells. Although the inhibition was not complete, the extent of inhibition of FITC-RAMEB uptake was similar to that of Lucifer Yellow (Fig. 9).

## Discussion

In the present study we investigated the permeability and cellular uptake of the fluorescent methyl-β-cyclodextrin in intestinal Caco-2 cells. The available data regarding the absorption and oral bioavailability of cyclodextrins is very limited and there are no Caco-2 permeability values in the literature. In early studies of intestinal absorption of ^14^C-labelled β-cyclodextrin in rats only 5% of the administered activity could be detected in the blood. It was concluded that β-cyclodextrin was not absorbed from the stomach and the small intestine, and the low absorption was explained with the amylase action: only the open-chain dextrins and the glucose formed from cyclodextrins were absorbed [21]. According to recent publications the oral bioavailability of HPBCD is less, than 0.03% and it is approximately 0.3% for β-cyclodextrin [22], while RAMEB has an oral bioavailability of about 12% in rats, [23].

Our permeability results with FITC-RAMEB are in accordance with the low intestinal absorption of cyclodextrins, as the P_app_ values were 3.35±1.29×10^−8^ and 4.23±1.46×10^−8^ cm/s for FR and FRR treatments, respectively. These data are also in agreement with the permeability results of natural cyclodextrins (α-, β-, and γ-cyclodextrin) on pulmonary Calu-3 cell layers, which were in the same order of magnitude [24].

Methylated cyclodextrins are also used for membrane cholesterol depletion in 5–10 mM concentration [4], [25] and can enhance the penetration of drugs [10]. 0.05 mM cyclodextrin presumably does not affect membrane cholesterol significantly, since it is 1/100 of the usually applied concentration. At 5 mM RAMEB concentration we did not observe cytotoxicity on Caco-2 cells previously [5], therefore we investigated the effect of 5 mM RAMEB on FITC-RAMEB permeability and the TEER of the monolayers. No significant difference could be observed on permeability and resistance values of FR and FRR treatments (p>0.05), and 5 mM RAMEB had no effect on the permeability of the monolayer.

Examining the fluorescence of the cells at the end of the permeability experiments we found that a significant amount of FITC-RAMEB accumulated in the cell layers. These cyclodextrins could not be removed by extensive washing. We investigated the time-dependence of FITC-RAMEB accumulation in the monolayers and as Fig. 3 shows Caco-2 cells successively accumulated both FR and FRR up to 120 min of the experiment. To reveal the fate of the accumulated FITC-RAMEB we loaded the cells with 0.5 mM FITC-RAMEB solution, using 10 time higher concentration than in permeability studies. This resulted in 10 time higher accumulation, but the permeability of FITC-RAMEB did not increase (2.28±0.34×10^−8^ cm/s). After 120 minutes, the release of the accumulated cyclodextrins was followed both in apical and basolateral directions. Interestingly FITC-RAMEB appeared in both the apical and basal chambers, but the majority of the accumulated cyclodextrin was released to the apical direction. Only 7.4% of the accumulated FITC-RAMEB reached the basal chamber.

These results indicated that although the intestinal Caco-2 monolayer is an almost impermeable barrier for the cyclodextrin molecules, the cells are able to take up cyclodextrins from solutions with a mechanism different from simple diffusion. Studies on Calu-3 monolayers suggested that cyclodextrins traverse these monolayers by paracellular route, although transcytosis could not be excluded [24]. Recent publications revealed that certain cell types are able to internalize cyclodextrins by endocytosis [16], [17], [18]; therefore we investigated the intracellular localization of FITC-RAMEB by confocal microscopy. Fig. 5 and 6 show that FITC-RAMEB is able to enter into the cytoplasm of both undifferentiated and differentiated Caco-2 cells. Since the labeled cyclodextrin was localized in vesicles in the cytoplasm, the possibility of endocytosis was investigated hereafter. In the cell membrane RFP-Rab5a fusion protein and FITC-RAMEB showed colocalization. Rab5 is a key organizer of early endosomes, but it cannot be detected in late endosomes [20]. In our confocal microscopy images Rab5 and FITC-RAMEB did not exhibit colocalization in vesicles in deeper layers. The colocalization of RFP-Rab5a and FITC-RAMEB suggests that endosome formation is involved in the initiation of cyclodextrin internalization.

Endocytosis has two major routes, phagocytosis and pinocytosis or fluid-phase uptake. Fluid-phase endocytosis, which requires the cargo molecules to be dissolved, can be subdivided into macropinocytosis, clathrin-mediated, caveolin-mediated and clathrin- and caveolin-independent endocytosis [26]. The widely used marker of macropinocytosis is Lucifer Yellow [27], [28], [29]. Flow cytometry analyses revealed that in Caco-2 cells Lucifer Yellow was internalized in a concentration dependent manner and its uptake could be inhibited at 0°C [28], [29]. FITC-RAMEB showed similar cellular uptake: at 37°C accumulated in the cells as a function of concentration, while at 0°C FITC-RAMEB uptake was diminished. On the other hand lipophilic calcein AM showed the same cellular accumulation at 0°C and 37°C, as it rapidly permeated the lipid membrane [30], and the intracellular accumulation was not inhibited by cooling. The macropinocytosis inhibitor rottlerin [27] had similar inhibitory effect on FITC-RAMEB and LY accumulation. These results indicate, that in Caco-2 cells macropinocytosis is involved in the entry of FITC-RAMEB. It also explains why the majority of the accumulated FITC-RAMEB was released to the apical direction. It was demonstrated in human epidermoid A431 cells, that macropinosomes recycle their content to the cell surface [31]. It seems that the same mechanism could be observed in Caco-2 cells, as the mechanism of internalization was macropinocytosis, the majority of accumulated cyclodextrin was guided to the apical cell surface. Nevertheless, the total recycling of the internalized cyclodextrin molecules took at least one hour, which means that this process prolongs the contact between cylodextrins or cyclodextrin-drug complexes and the membrane of macropinosomes. It is important to note, that other endocytotic mechanisms should be also taken into consideration. Previous studies implicated fluid-phase endocytosis and clathrin-dependent endocytosis [16], [17], [18] for the mechanism of cyclodextrin internalization. Nevertheless, phagocytosis could be also a possibility for cyclodextrin internalization in concentrated cyclodextrin solutions. It is reported that at high concentrations, natural β-cyclodextrin [32] and the fluorescent tetraamino rhodaminyl hydroxypropyl-β-cyclodextrin [33] form large, nano-sized aggregates in water. However, the substitution of OH groups with methyl groups on the cyclodextrin ring inhibits the aggregation of RAMEB and at 12 mM no aggregation was observed [32]. In this study 0.05 mM FITC-RAMEB was applied alone or in combination with 5 mM RAMEB, which is 40–240 times lower cyclodextrin concentration than what was found to form aggregates above, thus phagocytosis can be excluded from among the possible mechanisms of cyclodextrin uptake.

In summary, our results on Caco-2 cells are in accordance with earlier findings, the cellular internalization of water soluble FITC-RAMEB is governed by fluid phase endocytosis in intestinal Caco-2 cells. It is hard to predict the quantitative importance of this mechanism. Even if permeability data are suitable to value the extent of absorption of cyclodextrins, it is difficult to quantify the amount of continuously internalized and released cyclodextrins with this setup of the model. The intestinal absorptive surface area relative to the volume of the gut is much bigger than the surface area of Caco-2 monolayers and on the other hand the peristaltic movement should be also considered. Thus the extent of internalization can be much higher in vivo, even if cyclodextrins are released back to the lumen of the gut and as the process can be continuous along the small intestine its efficiency can be much higher.

## Conclusions

Cyclodextrins are used to increase solubility, bioavailability and stability of poorly water-soluble drugs. Our results demonstrate for the first time that randomly methylated-β-cyclodextrins can enter into intestinal epithelial cells by endocytosis. This process can contribute to the enhancement of the intestinal delivery and bioavailability of drugs by cyclodextrins in several ways. It can help to overcome the intestinal membrane barrier, the endosome formation increases the contact surface area between the cyclodextrin-drug complexes and the cell membrane and prolongs the retention time of cyclodextrins in the epithelial cells. Since this study has demonstrated the role of macropinocytosis in the uptake of methylated-β-cyclodextrin in intestinal cells, this mechanism merits further investigations in connection with drug absorption mediated by cyclodextrins.

## Supporting Information

Figure S1 **Colocalization of RFP-Rab5a with FITC-RAMEB, cell nucleus and cell membrane.** RAMEB shows high degree of colocalization with Rab5a immediately below the cell surface membrane of two connected cells at 2 minutes incubation (R = 0.93). Caco2 cells attached to surface of coverslip were transiently transfected by Rab5a (red) tagged by red fluorescent protein (RFP), treated by Fitc-RAMEB (green) for 2 minutes, and fixed. Before imaging surface membrane and nuclei were labeled by CellMask (dark blue) and DAPI (cyan), respectively, for 5 minutes. Panel A shows colocalization of Fitc-RAMEB and RFP-Rab5a; panel B indicates colocalization of DAPI and RFP-Rab5a as negative control (R = −0.39) and panel C specifies colocalization of surface membrane (CellMask) and RFP-Rab5a as positive control (R = 0.95) at a confocal image section crossing RAMEB granules (white areas in panel A right side, section thickness is 1.5 micrometer). Right panels show two channel images of signals tested for colocalization (bar is 10 micrometer), while corresponding left panels show two parameter histograms of signals of the two channels. White areas in right side images were chosen by setting channel signals above thresholds indicated by red signs on scales of corresponding left side two parameter histograms. R indicates Pearson correlation coefficients calculated in images at location of the white areas. In two parameter histograms the highest colocalization between tested channels would be indicated by a 45° diagonal line corresponding to R = 1 (in left panels of A and C R is close to this value), while a 135° diagonal line would indicate a negative correlation (left panel of B).
(TIF)

Figure S2 **Colocalization of FITC-RAMEB with RFP-Rab5a in the function of the time.** Colocalization of RAMEB and Rab5a was monitored in time during the endocytosis process. The highest average colocalization (0.76±0.01) was measured at 2 minutes after initiation of the endocytosis at 37°C. In later time points, at 5, 10, 20 and 30 minutes R was dropped to a lower but still significant value (R = 0.5–0.6). R, Pearson correlation coefficient was measured in region of interests (ROI) set to those locations where RAMEB granules were observed in confocal sections (one section was 1.5 micrometer thick). Pattern of the intracellular localization of colocalized molecules also changed in time. At 2 minutes colocalization was either dispersed in the surface membrane of cell or in the cytoplasm close to surface membrane. At later time points RAMEB granules moved closer to cell nuclei with lower, but still significant R for Rab5a colocalization (means±SD).
(TIF)
