# Supplementary figures and images for: Fluorescently Labeled Methyl-Beta-Cyclodextrin Enters Intestinal Epithelial Caco-2 Cells by Fluid-Phase Endocytosis
Source: PLoS One. 2014 Jan 8;9(1):e84856. doi: 10.1371/journal.pone.0084856 (PMC3885658; doi:10.1371/journal.pone.0084856)

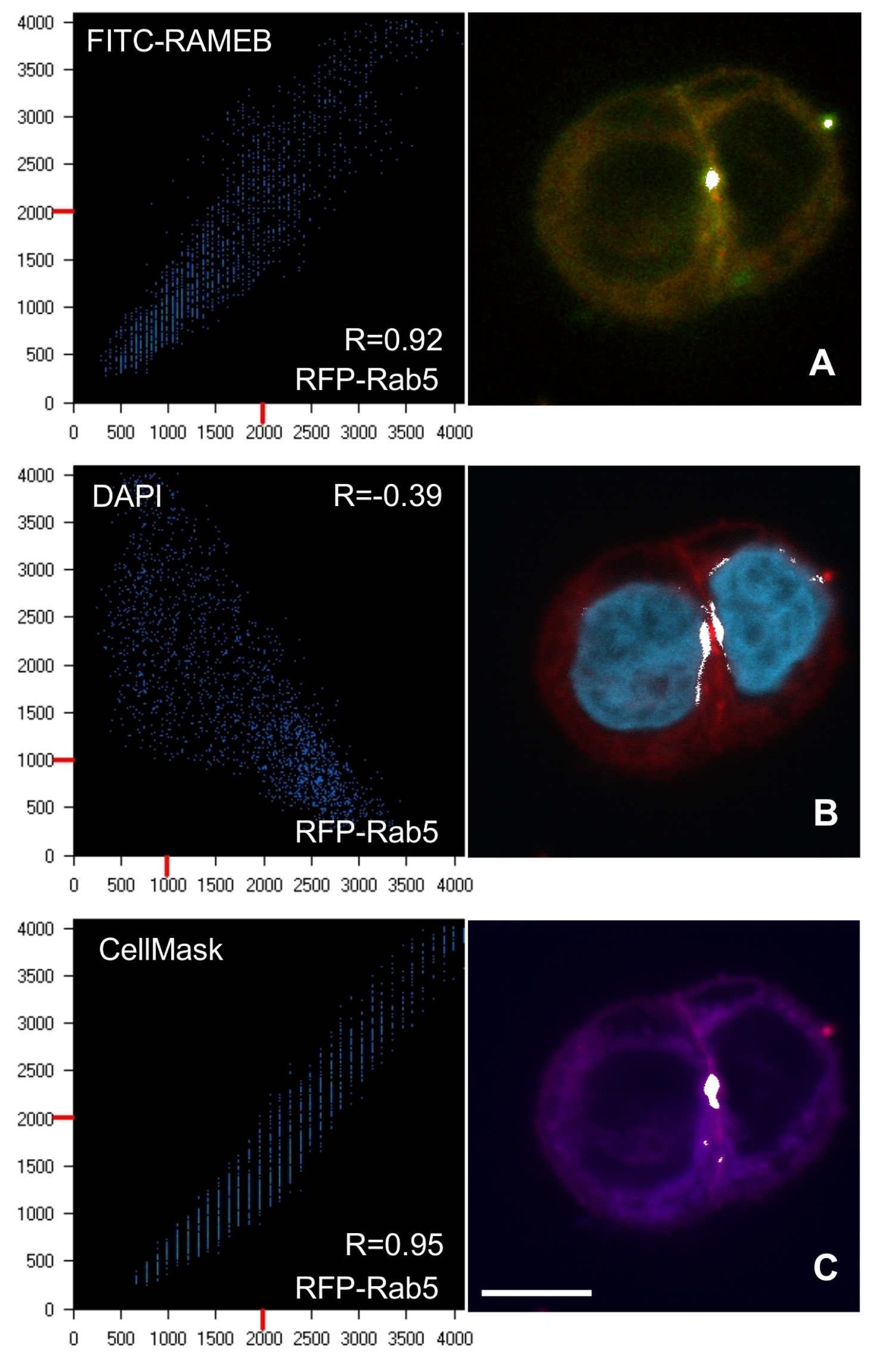

Supplement: Figure S1 — Colocalization of RFP-Rab5a with FITC-RAMEB, cell nucleus and cell membrane. RAMEB shows high degree of colocalization with Rab5a immediately below the cell surface membrane of two connected cells at 2 minutes incubation (R = 0.93). Caco2 cells attached to surface of coverslip were transiently transfected by Rab5a (red) tagged by red fluorescent protein (RFP), treated by Fitc-RAMEB (green) for 2 minutes, and fixed. Before imaging surface membrane and nuclei were labeled by CellMask (dark blue) and DAPI (cyan), respectively, for 5 minutes. Panel A shows colocalization of Fitc-RAMEB and RFP-Rab5a; panel B indicates colocalization of DAPI and RFP-Rab5a as negative control (R = −0.39) and panel C specifies colocalization of surface membrane (CellMask) and RFP-Rab5a as positive control (R = 0.95) at a confocal image section crossing RAMEB granules (white areas in panel A right side, section thickness is 1.5 micrometer). Right panels show two channel images of signals tested for colocalization (bar is 10 micrometer), while corresponding left panels show two parameter histograms of signals of the two channels. White areas in right side images were chosen by setting channel signals above thresholds indicated by red signs on scales of corresponding left side two parameter histograms. R indicates Pearson correlation coefficients calculated in images at location of the white areas. In two parameter histograms the highest colocalization between tested channels would be indicated by a 45° diagonal line corresponding to R = 1 (in left panels of A and C R is close to this value), while a 135° diagonal line would indicate a negative correlation (left panel of B). (TIF) [file pone.0084856.s001.tif]

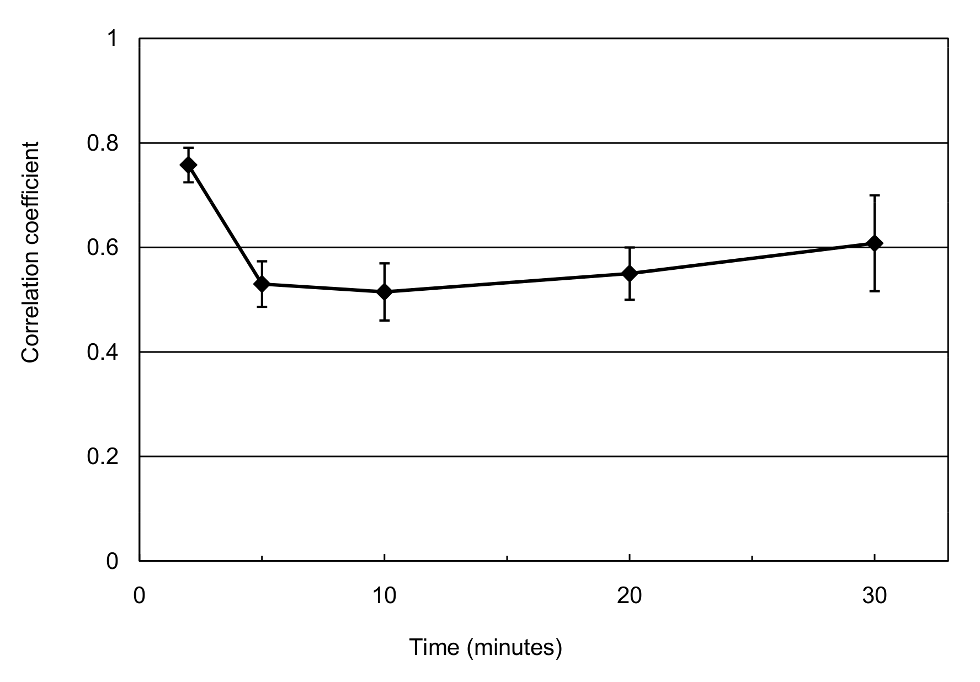

Supplement: Figure S2 — Colocalization of FITC-RAMEB with RFP-Rab5a in the function of the time. Colocalization of RAMEB and Rab5a was monitored in time during the endocytosis process. The highest average colocalization (0.76±0.01) was measured at 2 minutes after initiation of the endocytosis at 37°C. In later time points, at 5, 10, 20 and 30 minutes R was dropped to a lower but still significant value (R = 0.5–0.6). R, Pearson correlation coefficient was measured in region of interests (ROI) set to those locations where RAMEB granules were observed in confocal sections (one section was 1.5 micrometer thick). Pattern of the intracellular localization of colocalized molecules also changed in time. At 2 minutes colocalization was either dispersed in the surface membrane of cell or in the cytoplasm close to surface membrane. At later time points RAMEB granules moved closer to cell nuclei with lower, but still significant R for Rab5a colocalization (means±SD). (TIF) [file pone.0084856.s002.tif]
